# Assessment of a Polygenic Risk Score in Screening for Prostate Cancer

**DOI:** 10.1056/NEJMoa2407934

**Published:** 2025-04-10

**Authors:** Jana K McHugh, Elizabeth K Bancroft, Edward Saunders, Mark N Brook, Eva McGrowder, Sarah Wakerell, Denzil James, Reshma Rageevakumar, Barbara Benton, Natalie Taylor, Kathryn Myhill, Matthew Hogben, Netty Kinsella, Aslam A Sohaib, Declan Cahill, Stephen Hazell, Samuel J Withey, Naami Mcaddy, Elizabeth C Page, Andrea Osborne, Sarah Benafif, Ann-Britt Jones, Dhruv Patel, Dean Y Huang, Kaljit Kaur, Bradley Russell, Ray Nicholson, Fionnuala Croft, Justyna Sobczak, Claire McNally, Fiona Mutch, Samantha Bennett, Lenita Kingston, Questa Karlsson, Tokhir Dadaev, Sibel Saya, Susan Merson, Angela Wood, Nening Dennis, Nafisa Hussain, Alison Thwaites, Syed Hussain, Imran Rafi, Michelle Ferris, Pardeep Kumar, Nicholas D James, Nora Pashayan, Zsofia Kote-Jarai, Rosalind A Eeles, for the BARCODE1 Steering Committee and Collaborators, Antonis Antoniou, Audrey Ardern-Jones, Nicholas van As, Hywel Bowen-Perkins, Mark Brook, Declan Cahill, Anthony Chamberlain, David Dearnaley, Michelle Ferris, Steve Hazell, Denzil James, Kaljit Kaur, Vincent Khoo, Netty Kinsella, Zsofia Kote-Jarai, Pardeep Kumar, Eva McGrowder, Claire McNally, Christos Mikropoulos, Kenneth Muir, Holly Ni Raghallaigh, Judith Offman, Elizabeth Page, Nora Pashayan, Imran Rafi, Reshma Rageevakumar, Edward Saunders, Sibel Saya, Aslam Sohaib, James Taylor, Sarah Wakerell, The BARCODE1 Study Independent Data Monitoring Committee, Stephen Duffy, Dina Patel, John McGrath, Susan Wallace, Collaborators, David Nicol, Chris Ogden, Alan Thompson, Christopher Woodhouse

**Affiliations:** Centre for Cancer Genetic Epidemiology, University of Cambridge, UK; Royal Marsden NHS Foundation Trust, UK; Royal Marsden NHS Foundation Trust, UK; Primary Care Research Network (Kent, Surrey and Sussex); Institute of Cancer Research, UK; Royal Marsden NHS Foundation Trust, UK; Institute of Cancer Research, UK; Institute of Cancer Research, UK; Royal Marsden NHS Foundation Trust, UK; NHS Trust; Royal Marsden NHS Foundation Trust, UK; Institute of Cancer Research, UK; Royal Marsden NHS Foundation Trust, UK; Royal Marsden NHS Foundation Trust, UK; Royal Marsden NHS Foundation Trust, UK; Institute of Cancer Research, UK; Royal Marsden NHS Foundation Trust, UK; Institute of Cancer Research, UK; Institute of Cancer Research, UK; Institute of Cancer Research, UK; Royal Surrey NHS Foundation Trust, UK; University of Manchester, UK; Institute of Cancer Research, UK; Centre for Prevention, Detection and Diagnosis, Queen Mary University of London, UK; Institute of Cancer Research, UK; Centre for Cancer Genetic Epidemiology, Department of Public Health and Primary Care, University of Cambridge, UK; Institute for Medical and Biomedical Education, St George’s, University of London; Institute of Cancer Research, UK; Institute of Cancer Research, UK; Institute of Cancer Research, UK; University of Melbourne; Royal Marsden NHS Foundation Trust, UK; Patient Representative, UK; Institute of Cancer Research, UK; Wolfson Institute of Population Health, Queen Mary University of London, UK; UK NEQAS Immunology, Immunochemistry & Allergy, Shefield, UK; Royal Devon University Healthcare NHS Foundation Trust; University of Exeter Medical School, UK; Department of Health Sciences, University of Leicester, UK; Royal Marsden NHS Foundation Trust, UK; 1 The Institute of Cancer Research, Sutton, United Kingdom; 2 The Royal Marsden NHS Foundation Trust, London, United Kingdom; 3 Translational Oncology & Urology Research (TOUR) Centre for Cancer, Society, and Public Health, King's College London, United Kingdom; 4 University College London Hospitals NHS Foundation Trust, London, United Kingdom; 5 InHealth Limited, InHealth Group HQ, High Wycombe, United Kingdom; 6 The University of Melbourne, Melbourne, Australia; 7 St George's University of London, London, United Kingdom; 8 Lane End Medical Group, London, United Kingdom; 9 University of Cambridge, Cambridge, United Kingdom

**Keywords:** prostate cancer, targeted prostate cancer screening, polygenic risk score, genetic risk, prostate specific antigen test

## Abstract

**Background:**

The incidence of prostate cancer (PrCa) is increasing. Screening by assay of prostate-specific antigen (PSA) has a high false-positive rate. Genome wide association studies have identified common germline variants, which can be used to calculate a polygenic risk score (PRS) associated with PrCa risk.

**Methods:**

The BARCODE1 study recruited persons aged 55 to 69 yrs via primary care in the UK. PRS were derived from 130 PrCa risk variants in germline DNA extracted from saliva. Participants with a PRS >90^th^ centile were invited for PrCa screening using multiparametric Magnetic Resonance Imaging (MRI) and transperineal biopsy, irrespective of PSA result.

**Results:**

Of 40,292 persons invited to participate, 8,953 (22.2%) expressed interest in participating and 6,393 had their PRS calculated, of whom 745 (11.7%) had a PRS >90^th^ centile and were invited for screening. Of these 745 participants, 468 underwent MRI and prostate biopsy; PrCa was detected in 187 (40.0%) of them. Median age at diagnosis was 64 yrs (range 57 to 73 yrs). Using NCCN criteria (2023), 103 (55.1%) cancers were of intermediate or high risk and so required treatment; 73 (70.9%) of these cancers would not have been detected using the UK PrCa diagnostic pathway. Of the 187 cancers, 40 (21.4%) were ‘Intermediate Unfavorable’/’High’/’Very High-Risk’.

**Conclusions:**

Risk stratification by PRS of 6,393 persons led to the detection of PrCa requiring clinical management in 103 participants, of whom 73 (70.9%) would have been missed using the standard diagnostic pathway used in the UK.

(Funded by European Research Council Seventh Framework Programme, and others. ClinicalTrials.gov, NCT03857477)

## Introduction

Prostate cancer (PrCa) is a considerable health burden worldwide; it is the most common cancer in people assigned male at birth, after skin cancer, and caused 375,000 deaths in 2020^1^. There is no international population-based screening programme for the early detection of PrCa. The clinical utility of prostate-specific antigen (PSA) assay for monitoring PrCa progression is indisputable, but its use as a screening tool is debated due to the potential harms outweighing the benefits. It has been evaluated in two large, randomised studies: The Prostate, Lung, Colorectal and Ovary (PLCO) study^2^ and the European Randomised Study of Screening for Prostate Cancer (ERSPC).^3^ Criticisms of PSA-screening include a high false-positive rate, over-diagnosis, complications associated with prostate biopsies and overtreatment of low-grade disease, but the ERSPC showed that after 22 years, mortality was approximately 30 percentage points higher in the control group compared with men who were offered screening through biennial PSA testing.^3^

For persons diagnosed with PrCa at stage I/II, the 5-year survival rate is almost 100%; for persons diagnosed with stage IV disease, it is 50%.^4^ Therefore, an effective screening tool to detect early-stage, clinically significant PrCa (csPrCa) is urgently needed. Research is focussed on Magnetic Resonance Imaging (MRI)–based screening,^5–7^ biomarkers^8,9^ and modelling multiple risk factors.^10,11.^

Age and family history are established PrCa risk factors. PrCa is highly heritable, with 58% heritability observed in twin studies.^12^ A small proportion of germline genetic risk is caused by rare pathogenic variants in DNA-repair genes (e.g. *BRCA1* and *BRCA2*), and a greater proportion is due to the combined effect of multiple low-risk variants, called single nucleotide polymorphisms (SNPs), from which one’s polygenic risk score (PRS) can be calculated.^13^ Following a pilot study,^14^ the BARCODE1 study was prospectively designed to test the performance of PRS in a general-population PrCa-screening programme in stratifying people for targeted screening.

Here we report the baseline outcomes of the BARCODE1 study. We report on the uptake, cancer detection, and positive predictive value (PPV) of MRI and biopsy, and the proportion of those in the PRS >90^th^ centile for PrCa risk who were diagnosed with PrCa.

## Methods

### Study Design, Setting and Participants

BARCODE1 is a prospectively-designed, single-arm study that received approval from the London-Chelsea Research Ethics Committee (reference:18/LO/2166) and the Health Research Authority (reference:257684).

Recruitment was coordinated through 69 primary-care centres from three Clinical Research Networks (Kent, Surrey and Sussex; South London; and the Thames Valley and South Midlands) between March and July, in 2019. Patient databases were screened, and eligible individuals invited by letter. Eligibility included people assigned male at birth, aged 55-69 yrs, of European ancestry (self-reported), no personal history of PrCa, not currently under investigation for suspected PrCa, no prostate biopsy within 12 months, and no known contraindications to MRI or biopsy (Table S1, Supplementary information). Interested individuals completed a health-screening questionnaire and provided written informed consent and a postal saliva sample for genetic analysis.

DNA extraction was carried out at Yourgene Health, UK. Extracted DNA was sent to Affymetrix (Thermo Fisher Scientific Inc., USA) for genotyping using a custom-designed high-throughput assay (Eureka™ myDesign Genotyping Panel.^14^ The panel consisted of 130 European-ancestry PrCa risk SNPs (Table S2), and has been validated for use only in people of European ancestry.^15^ The PRS of each participant was calculated using the sum of weighted alleles for the 130 SNPs.

BARCODE1 participants with a PRS ≥90^th^ centile (based on a reference population from the ProtecT study (Prostate Testing for Cancer and Treatment))^16^ were referred to a cancer centre for genetic-risk counselling. This counselling involved a discussion about the meaning of the PRS results with experienced clinicians. Participants were offered a PSA, multiparametric MRI and transperineal biopsy. The InHealth Group performed MRIs through 7 diagnostic centres. These were reported according to PI-RADS v2.1^17^ by one of two consultant radiologists, both considered expert according to ESUR consensus guidance. Biopsies were performed using a transperineal approach under local anaesthesia. When lesions were identified through MRI, an MRI-guided targeted biopsy was undertaken. Histopathology was reported by a urological consultant histopathologist. Participants diagnosed with PrCa were managed in accordance with NICE guidelines.^18^ Cancers were defined using Gleason Score and the NCCN 2023 criteria (Very Low, Low, Intermediate Favourable, Intermediate Unfavourable, High or Very High Risk of metastasis).^19^ Cancers were “clinically significant” if Gleason Score ≥3+4. Participants with negative biopsies were then screened annually (Figure S1) for 5 years.

#### Sample size

A sample of 5,000 participants was required to identify approximately 500 individuals with a PRS ≥90^th^ centile. Assuming the 130 SNPs interact log additively, polygenic variance was estimated to be 0.52 (by calculating first the variance explained by each SNP and then summing up the contributions (methods in^20^). On the basis of this total polygenic variance, and using previously described methodology^21^, we assumed that those in the top 10% of the risk distribution would account for 29% of all PrCa cases.

#### Statistical analysis

Descriptive statistics were used to analyse test uptake, proportion of participants accepting biopsy, cancer detection rate, age at diagnosis, PPV of prostate biopsy, PPV of PSA, PPV of MRI (presence of PI-RADS 3-5 lesion) and stratification of tumours using Gleason Score and NCCN 2023 risk classification. Statistical analysis was conducted by MNB, EKB, JKM, NP. Full copies of the statistical analysis and protocol are available on NEJM.org.

Logistic regression modelled the association of biopsy outcome with age, family history (defined as any first or second-degree relative with PrCa), PSA, PI-RADS and PSA density (PSA density is calculated as the PSA level (ng/mL) divided by the volume of the prostate (mL), with the aim of taking into account that a larger prostate gland may produce more PSA. Biopsy outcome was modelled for any PrCa and for csPrCa. Univariable models were evaluated for each variable of interest. Models were then developed to include age and family history (as established risk factors) along with exhaustive combinations of PSA and PI-RADS. AUC was calculated for each model.

Ten-year absolute risk was calculated for each participant using the iCARE package,^22^ incorporating age-specific incidence rates of PrCa^4^, competing mortality rates,^23^ relative risk from family history,^24^ and PRS.

We estimated the probability of overdiagnosis as the probability that screen-detected cancer would have taken longer than the remaining lifetime to progress to clinical cancer^24^. We derived age-specific mean sojourn time (mean sojourn time is defined as the length of time between when a condition can be detected by screening and when it would present clinically) as the weighted sum of the mean sojourn time for tumours with Gleason Score <7 and Gleason Score ≥7 for each scenario^25^. We derived the expected remaining lifetime by age from the UK national life table for 2020-2022.^23^ We calculated the probability of overdiagnosis as the probability of the mean sojourn time to be greater than the expected remaining lifetime (see supplementary information S8).^25^

## Results

### Study population

**Figure 1:**
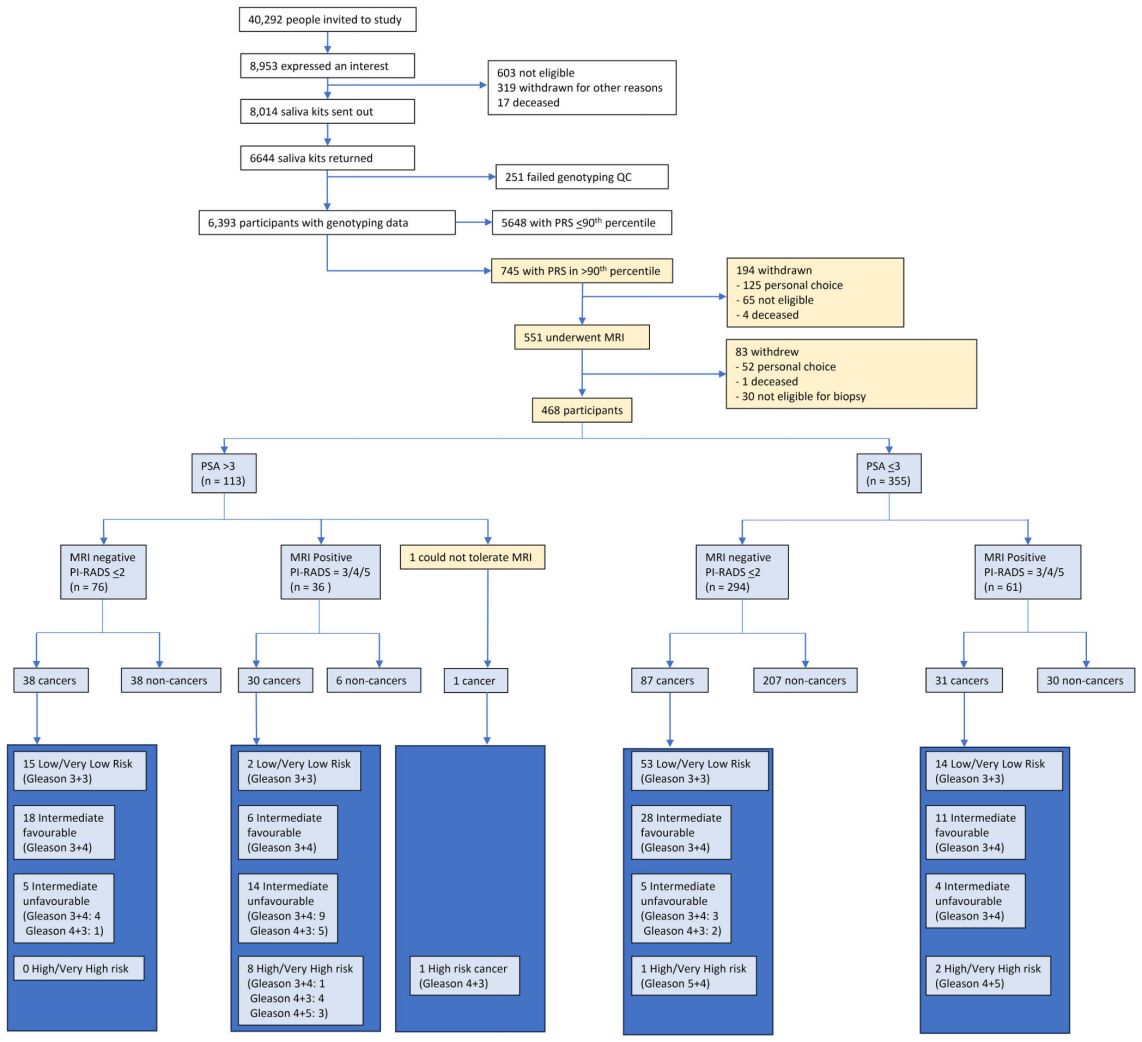
is a consort diagram that summarises the participant pathway through the BARCODE1 study from expression of interest through to biopsy outcome. PRS = Polygenic Risk Score, QC = Quality Control; PSA = prostate-specific antigen, MRI = Magnetic Resonance Imaging, PI-RADS = Prostate Imaging Reporting and Data System. Cancers are classified by the NCCN (2023) Risk Groups (very low, low, intermediate favourable, intermediate unfavourable, high or very high risk).

From 40,292 participants invited, 8,953 expressed an interest in BARCODE1 (22.2%; Figure 1). 6,393 participants were genotyped, and 745 had a PRS ≥90^th^ centile; 468 participants (62.8%) accepted MRI and biopsy. 177 withdrew through personal choice, 95 were withdrawn by the study team (Table S3), including 8 who had PrCa diagnosed prior to receiving their PRS and 5 died before study completion (owing to unrelated causes). The mean age at enrolment was 61.2 yrs and 20.9% reported a family history of PrCa (Table S4).

### Prostate Cancer Detection Rates and Cancer Characteristics

We detected PrCa in 187 (40%) of the 468 participants who underwent biopsy. The median age at diagnosis was 64 yrs (range 57-73 years) (Figure 1, Tables 1-2). The mean number of cores taken at biopsy was 12.7 (range 5-18).

Of the 187 cancers detected by biopsy, 103 (55.1%) were Gleason Score ≥7 and classified as ‘Intermediate’ or more advanced, according to NCCN classification (2023); 40 (21.4%) were 'Intermediate Unfavourable’/ ‘High’/ ‘Very High’ risk (by NCCN classification).

### Cancer Detection and PSA level

Median PSA concentration at diagnosis was 2.1 ug/L (range 0.25-274 ug/L). Of the cancers,118/187 (63.1%) had PSA <3.0ug/L; 51 (43.2%) were Gleason Score ≥7. Of the cancers detected in the participants with a PSA >3.0ug/L, 52/69 (75.4%) had Gleason Score ≥7. Table S5 shows the breakdown of cancers detected stratified by PSA level.

### Cancer Detection and MRI Characteristics

Overall, 97 participants had a PI-RADS score ≥3 and underwent a targeted biopsy. Of the 43 persons with a PI-RADS 3 lesion, 19 had cancer detected at biopsy (9 had Gleason Score ≥7). Of the 54 persons with PI-RADS ≥4 lesions, 42 (77.8%) had cancer detected at biopsy (36 had Gleason Score ≥7, 25 classified as ‘Intermediate Unfavourable’/’High’ or ‘Very high-risk’ disease). Of the 370 persons with negative results (PI-RADS <2) on MRI, 125 had cancer detected through biopsy, of which 57 were Gleason Score ≥7.

**Figure 2:**
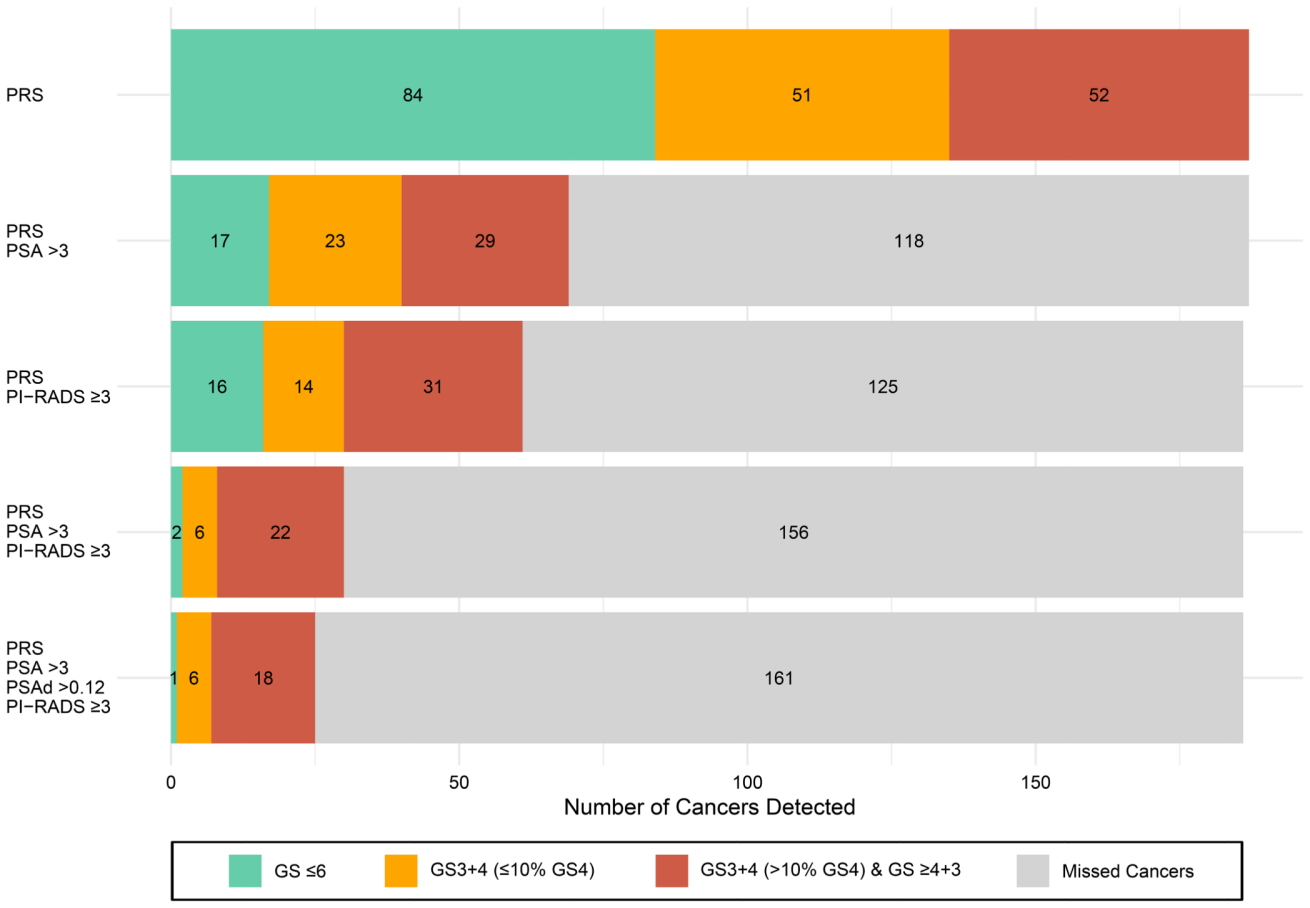
The top bar shows all PrCa detected using PRS alone. The cancers are divided into 3 groups: Gleason Score ≤6 shown in green, Gleason Score 3+4 with ≤10% Gleason pattern 4 present shown in orange and Gleason Score 3+4 with >10% Gleason pattern 4 shown in red. The second bar shows the cancers detected if a PSA threshold of >3.0ug/l were used to stratify. The third bar shows the cancers detected if an MRI PI-RADS threshold of ≥3 were used to stratify. The fourth bar shows the cancers detected if a PSA threshold of >3.0ug/L and MRI PI-RADS score of ≥3 were used to stratify. The final bar shows the cancers detected if a PSA threshold of >3.0ug/L, PSA density of >0.12ng/ml/cc and MRI PI-RADS score of ≥3 were used to stratify. For the groups that include MRI, the total number of cancers evaluated were 186 of the overall 187, as one patient was unable to undergo MRI due to claustrophobia.

Of the 187 participants diagnosed with PrCa, 100 had either a high PSA level (>3.0 ug/L) or a PI-RADS 3-5 lesion; only 30 (16.0%) had both a high PSA level and a PI-RADS 3-5 lesion, that together comprise the standard criterion in the traditional management pathway for progressing to prostate biopsy (Figure 2, Table 2). Of the 40 diagnosed ‘Intermediate Unfavourable’/ ‘High risk’ cancers, 17 (42.5%) would have been missed using the standard criterion. The addition of PSA density did not add discriminatory value; 48 of the 186 individuals with PrCa had a PSA density ≥0.12ng/ml/cc.^26^

### Positive Predictive Values

The PPV of a PSA threshold >3.0 ug/L for detection PrCa of by biopsy (in the 468 participants who underwent biopsy) was 61.1% and that of MRI (PI-RADS 3-5) was 62.9% (Table S6).

### Logistic Regression

Univariable models for those participants in the top 10% of the PRS distribution showed that age and family history were not associated with ‘any PrCa’ and provided no discriminatory accuracy. However, they were strongly associated with csPrCa and provided some modest discriminatory accuracy. PSA level and PI-RADS score re were strongly associated with both cancer outcomes and provided strong discriminatory accuracy. PSA density did not add any discriminatory value.

The strongest performing model included age, family history, PSA level, and PI-RADS score m. Both PSA level and PI-RADS score were strongly associated with cancer outcome and provided good discrimination for any PrCa (AUC=0.69) and csPrCa (AUC=0.78) (Table S7).

Further stratification by PRS (i.e. 90^th^ vs 99^th^ centile) was not associated with cancer outcome and added little to any of the models described.

### Absolute Risk

**Figure 3:**
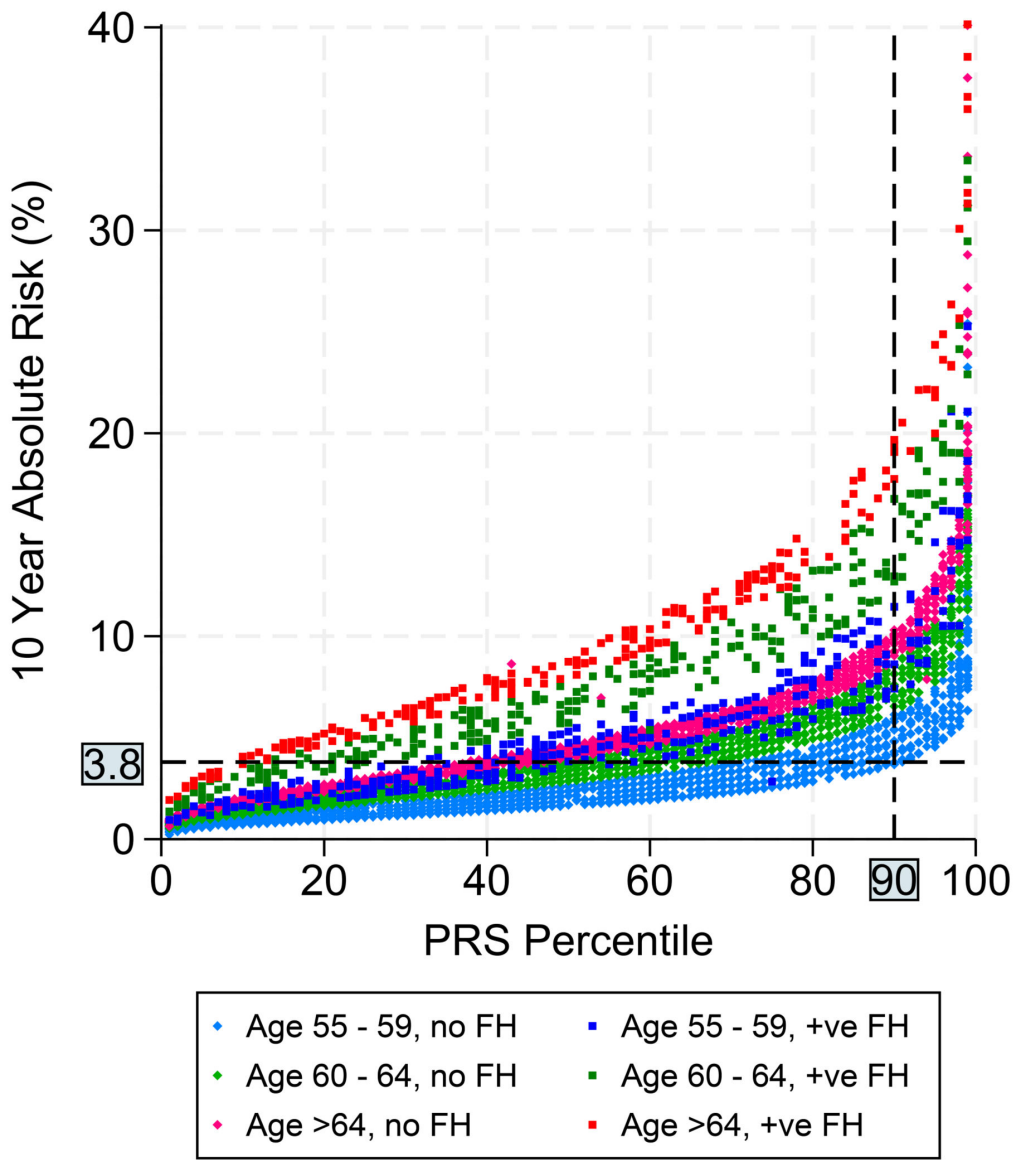
A threshold of 3.5%-4% ten-year absolute risk has been suggested as generating the greatest number of quality-adjusted life-years from risk-based screening

Figure 3 shows the 10-year absolute risk against PRS percentile, stratified by age and family history. In the figure those with PRS ≥90^th^ centile are almost all above the 3.8% (red horizontal line) 10-year absolute risk cut-off. Others that are above the cut-off in lower PRS centiles have a family history of PrCa. This figure highlights that PRS does not replace known risk factors but supplements them in risk stratification.

### Overdiagnosis

We estimated that 39 (20.8%) (range 9.7-33.9% for ages 55 to 74) of persons in ≥90^th^ centile PRS and with screen-detected cancer would be “overdiagnosed”: ie, their screen-detected PrCa would take longer than their remaining lifetime to progress to clinical cancer. If using a PSA threshold of >3.0ug/L only, 12 (17.2%) (range 7.0-21.0%) would be “overdiagnosed” and if using PI-RADS ≥3 lesion only, 15.6% (range 4.0-25.0%) would be “overdiagnosed” (Tables S8-S10). Had we screened the 187 participants (in whom we diagnosed cancer in this study) with the standard UK PrCa screening method, we would have avoided detecting 27 (26.7%) of participants with clinically insignificant cancer.

### Adverse Events

One (0.2%) participant had sepsis post-biopsy and required hospitalisation for intravenous antibiotics. Two participants (0.4%) had a urine infection <7 days post-biopsy that required oral antibiotics. One participant (0.2%) required temporary catheterisation immediately post-biopsy (Table S12).

## Discussion

Our results show that offering targeted screening to persons in the ≥90^th^ centile of genetic risk distribution as determined by PRS, resulted in the detection of PrCa requiring clinical management in 55.1% and radical treatment in 21.4% of these persons.

The current UK diagnostic pathway for suspected PrCa (PSA >2.5ug/L <50 years; >3.5ug/L 50-60 years and >4.5ug/L 60-70 years or abnormal digital-rectal examination^18^) results in referral for MRI. If a lesion is present, or there is other clinical concern, biopsy is indicated. If the participants of BARCODE1 had followed this pathway, 42.5% of the csPrCa would have been missed, and 26.7% of clinically insignificant PrCa would have been avoided.

It is notable that 40% of participants had PrCa detected at biopsy and 55% of these cancers had a Gleason Score ≥7. In the ERSPC, the decision on whether to biopsy rested on PSA level, 35.5% of the participants were found to have PrCa.^27^ When we restricted the analysis of BARCODE1 participants in the ≥90^th^ centile to only those with PSA >3.0ug/L, we found that 75.4% of cancers detected were Gleason Score ≥7. The PPV of PSA >3.0ug/L with respect to having biopsy-confirmed PrCa in the ERSPC was 24.1%. The PPV of the ≥90^th^ centile of BARCODE1 was 40%.

The STHLM3 screening study compared PSA (using a threshold of ≥3ug/L) with a combination of plasma biomarkers, 232 risk SNPs and clinical variables.^11^ In this study the AUC of PSA alone was 0.56; the AUC of PSA and the additional risk factors was 0.74. In BARCODE1, the AUC was 0.78 when combining PRS with age, family history, PI-RADS score and PSA. In STHLM3 it was difficult to assess the contribution of the SNP profile to the screening model;^11^ in contrast, BARCODE1 used PRS alone as a risk-stratification tool.

Studies have shown that combining multiparametric MRI with targeted biopsies of lesions improves detection of csPrCa (i.e. Gleason Score ≥7)^6,7,28,29^. However, real-world data indicate that up to 25% men with no lesion detected by MRI may have csPrCa on biopsy.^30^ BARCODE1 identified csPrCa in persons without MRI lesions, suggesting that for those with a PRS ≥90^th^ centile, prostate biopsy should be considered regardless of MRI outcome. However, nearly half the cancers diagnosed through biopsy alone would be predicted to have a Gleason Score of <7: so there is trade-off between minimizing the odds of overdiagnosis and missing csPrCa. Adding PSA density to our models did not improve detection of csPrCa^26^. The biology of PrCa may differ between persons who have a genetic predisposition to developing PrCa and those who do not. Further research is needed to determine the link between specific SNPs and PrCa aggressiveness.^31^

The IMPACT study targeted PrCa screening at people with pathogenic variants in *BRCA1* and *BRCA2*. Using a PSA of >3.0ug/L to indicate biopsy, the PPV of PSA screening was 36% overall and 48% for *BRCA2* carriers, compared with 61.1% in BARCODE1.^32^ A higher proportion of csPrCa was reported in the IMPACT study (61%) than in BARCODE1 (55%). The results of the IMPACT study led the European Association of Urologists to recommend screening for *BRCA2* carriers from age 40.^33^

A UK-based study has showed that using a 10-year absolute risk threshold of 3.5%-4% for developing PrCa in risk-based screening yields the greatest number of quality-adjusted life-years gained^34^. Figure 3 demonstrates that this threshold of absolute risk includes almost all those in the ≥90^th^ centile of PRS, or those with a family history of disease, or those of older age and not in the lower PRS categories. This supports the use of PRS together with established risk factors in screening for PrCa. Further study will be required to determine whether PRS could identify those at low risk who may benefit from a less intensive screening regimen and those who should be considered for further evaluation if the PSA is below commonly accepted thresholds and for a biopsy even if MRI is non-suspicious. A PRS can be carried out once in a person’s lifetime, as it does not change with age. In BARCODE1 all men were ≥55 years, and further evaluation of the timing of PRS and subsequent screening algorithms will be needed to assess trade-off of benefits, harms and cost-effectiveness.

Our estimate of overdiagnosis in BARCODE1 (15.6 to 20.8%) is similar to the overdiagnosis estimates in two PSA-based screening studies.^24,35^ Further screening will be key to ascertain PrCa incidence over time in currently unaffected high-risk individuals. Follow-up of the whole cohort will determine PrCa incidence and tumor characteristics for participants in the <90^th^ centile of the PRS and will enable an evaluation of the economic and clinical impact of using PRS as a risk-stratification tool within a PrCa-screening programme.

There is good evidence that active surveillance manages indolent PrCa at relatively low cost whilst detecting progression at a curable stage.^36^ Approximately 30-40% of individuals enrolled in active surveillance have disease progression, with those at higher genetic risk more likely to fall into this category.^37^ All but one of the persons with PrCas with Gleason Score 6 detected in BARCODE1 (comprising 44.4% of detected PrCas) are under active surveillance. While BARCODE1 may have led to some overdiagnosis, it has not led to overtreatment of indolent disease. BARCODE1 will follow up participants and report on rates of disease progression.

### Limitations

BARCODE1 had an uptake of 22%. The participant information emphasised the need for prostate biopsy in those identified as ‘high-risk’. Reluctance to undergo biopsy was the predominant reason (40.7%) for participants choosing to withdraw, both before and after MRI. Uptake and compliance were likely heavily affected by the COVID-19 pandemic which coincided with the roll-out of BARCODE1.

This was a self-selected homogenous population; participants were highly educated and largely from professional occupations. All participants were of European ancestry due to the limitations of the PRS at the time of study design and therefore not representative of the UK general population (Table S11). Genome-wide association studies have provided data on risk SNPs across diverse ancestral groups and research focussed on using genetic ancestry-specific PRS for risk-based screening is in progress. BARCODE1 provides a framework on which to build further research on the role of genetic risk in screening for cancer in persons of non-European ancestries. This includes those at higher risk of PrCa such as persons of Black African and Caribbean ancestry where lifetime risk in the UK is quoted as 1 in 4 compared with 1 in 8 for those of European ancestry (4). In future work it will be important to consider the role of both rare and common genetic variants in understanding genetic risk in PrCa in all ancestries.

Another limitation is the potential for selection bias for those with a family history of PrCa; such persons might be more likely to accept the invitation to join the study. However, only ~20% of participants reported having a family history, indicating family history of PrCa does not seem to have had a major impact on uptake in BARCODE1.

In summary, PRS within a population-based PrCa screening programme detected a high proportion of csPrCa (Gleason score ≥7) requiring treatment on national guidelines, compared with PSA or MRI-based screening programmes. To fully evaluate the implementation of PRS alongside established risk factors in a national screening programme, further research is required, including research into optimal age at which to obtain a PRS, tests of replication in people of non-European ancestry and an evaluation of economic impact.

## Supplementary Material

Supplement

## Figures and Tables

**Table 1:** Summary of cancer outcomes by PSA, MRI, PRS and family history

		No cancer		Any Cancer		Clinically Significant Cancer	
Age at biopsy, Med (IQR)		63	(60, 67)	64	(60, 68)	65	(60, 69)
PSA, Med (IQR)		1.4	(0.9, 2.3)	2.1	(1.3, 4.2)	3.1	(1.8, 6.3)
PRS centile, Med (IQR)		95	(92, 98)	95	(93, 99)	96	(93, 99)
PI-RAD, N (%)	1	7	(2.5)	1	(0.5)	1	(1.0)
	2	238	(84.7)	124	(66.7)	56	(54.9)
	3	24	(8.5)	19	(10.2)	9	(8.8)
	4	10	(3.6)	22	(11.8)	17	(16.7)
	5	2	(0.7)	20	(10.8)	19	(18.6)
Family History (1st or 2nd degree rels), N (%)	N	232	(82.6)	147	(78.6)	74	(71.8)
	Y	49	(17.4)	40	(21.4)	29	(28.2)

**Table 2:** Characteristics of the cancers detected and those that would have been missed stratified by PRS, PSA and/or MRI*

	PRS (top 10%)		PSA (>3)				MRI				PSA + MRI			
	Cancers, N	(%)	Cancers, N	(%)	Cancers Missed, N	(%)	Cancers, N	(%)	Cancers Missed, N	(%)	Cancers, N	(%)	Cancers Missed, N	(%)
Low/Very Low	84	(44.9)	17	(24.6)	67	(56.8)	16	(26.2)	68	(54.4)	2	(6.7)	82	(52.6)
Intermediate Favourable	63	(33.7)	24	(34.8)	39	(33.1)	17	(27.9)	46	(36.8)	6	(20.0)	57	(36.5)
Intermediate Unfavourable	28	(15.0)	19	(27.5)	9	(7.6)	18	(29.5)	10	(8.0)	14	(46.7)	14	(9.0)
High/Very High	12	(6.4)	9	(13.0)	3	(2.5)	10	(16.4)	1	(0.8)	8	(26.7)	3	(1.9)
**Total**	**187**		**69**		**118**		**61**		**125**		**30**		**156**	

* 1 participant with prostate cancer could not tolerate MRI
