# Supplementary material for: Assessment of a Polygenic Risk Score in Screening for Prostate Cancer
Source: N Engl J Med. Author manuscript; Available in PMC 2025 Apr 24. (PMC7617604; doi:10.1056/NEJMoa2407934)
Supplement: Supplement [file EMS202171-supplement-Supplement.pdf]

Supplementary Material Annex

Table of Contents	Page Number
BARCODE1 study Steering Committee	2
BARCODE1 study Collaborators	3
BARCODE1 study Collaborators: GP Practices	3-5
Figure S1: Study Design	6
Table S1: Eligibility Criteria	7
Table S2: List of 130 Risk SNPs used to calculate PRS	8
Table S3– Reasons for declining / withdrawing	9
Table S4: Sociodemographic characteristics of those in the top 10% of PRS	10
Table S5: Breakdown of cancers stratified by PSA level	10
Table S6: Summary of positive (PPV) and negative predictive values (NPV), sensitivity and specificity and area under the curve (AUC)	11
Table S7: Univariable and multivariable models	12
Table S8: Estimates of overdiagnosis	13
Supplementary Information 1: Overdiagnosis calculations	14
Table S9: Mean sojourn time estimates by Gleason score and age groups	14
Table S10: Expected remaining lifetime by age for men in the UK , 2020-2022	15
Table S11: Diversity and Representativeness Table: demographics of the UK population affected by prostate cancer	16
Table S12: Description of all adverse events in the BARCODE1 Study	17
Supplementary Information: Participant questionnaire used to collect demographic and health information	18-21

<u>The BARCODE1 Study Steering Committee:</u>

Prof Antonis Antoniou - Professor of Cancer Risk Prediction, Centre for Cancer Genetic Epidemiology, University of Cambridge, UK

Audrey Ardern-Jones – Senior Clinical Nurse Specialist in Cancer Genetics, Royal Marsden NHS Foundation Trust, UK

Prof Nicholas van As – Consultant Clinical Oncologist, Royal Marsden NHS Foundation Trust, UK

Dr Hywel Bowen-Perkins – GP; Member of Primary Care Research Network (Kent, Surrey and Sussex)

Dr Mark Brook – Biostatistician, Institute of Cancer Research, UK

Mr Declan Cahill – Consultant Urologist, Royal Marsden NHS Foundation Trust, UK

Mr Anthony Chamberlain - Research Assistant, Institute of Cancer Research, UK

Prof David Dearnaley – Professor of Uro-Oncology, Institute of Cancer Research, UK & Royal Marsden NHS Foundation Trust, UK

Dr Michelle Ferris – General Practitioner-Barnet Primary Care NHS Trust

Dr Steve Hazell – Consultant Histopathologist, Royal Marsden NHS Foundation Trust, UK

Denzil James – Project Manager and Sample Coordinator, Institute of Cancer Research, UK

Kaljit Kaur – Lead Nurse for Urology, Royal Marsden NHS Foundation Trust, UK

Dr Vincent Khoo – Consultant Clinical Oncologist, Royal Marsden NHS Foundation Trust, UK

Dr Netty Kinsella – Nurse Consultant for Uro-Oncology, Royal Marsden NHS Foundation Trust, UK

Dr Zsofia Kote-Jarai – Senior Staff Scientist, Institute of Cancer Research, UK

Mr Pardeep Kumar - Consultant Urologist, Royal Marsden NHS Foundation Trust, UK

Dr Eva McGrowder - Study Coordinator, Institute of Cancer Research, UK

Claire McNally – Clinical Nurse Specialist for Urology, Royal Marsden NHS Foundation Trust, UK

Dr Christos Mikropoulos – Clinical Research Fellow, Institute of Cancer Research, UK; now at Royal Surrey NHS Foundation Trust, UK

Prof Kenneth Muir – Professor of Cancer Epidemiology-University of Manchester, UK

Dr Holly Ni Raghallaigh - Clinical Fellow, Institute of Cancer Research, UK

Dr Judith Offman - Senior Lecturer, Centre for Prevention, Detection and Diagnosis, Queen Mary University of London, UK

Elizabeth Page – Study Coordinator, Institute of Cancer Research, UK

Dr Nora Pashayan – Professor of Epidemiology and Aging, Centre for Cancer Genetic Epidemiology, Department of Public Health and Primary Care, University of Cambridge, UK

Dr Imran Rafi – Reader Primary Care and Genomics, Institute for Medical and Biomedical Education, St George's, University of London

Reshma Rageevakumar – Research Assistant, Institute of Cancer Research, UK

Edward Saunders – Higher Scientific Officer, Institute of Cancer Research, UK

Dr Sibel Saya – Post-Doc Research Fellow and Genetic Counsellor, Institute of Cancer Research, UK and University of Melbourne

Dr Aslam Sohaib – Consultant Radiologist, Royal Marsden NHS Foundation Trust, UK

James Taylor – Patient Representative, UK

Sarah Wakerell – Higher Scientific Officer, Institute of Cancer Research, UK

The BARCODE1 Study Independent Data Monitoring Committee:

Prof Stephen Duffy (Chair), Wolfson Institute of Population Health, Queen Mary University of London, UK

Dr Dina Patel, Scientific Director, UK NEQAS Immunology, Immunochemistry & Allergy, Sheffield, UK

Mr John McGrath, Consultant Urologist, Royal Devon University Healthcare NHS Foundation Trust, Honorary Senior Lecturer, University of Exeter Medical School, UK

Dr Susan Wallace, Lecturer of Population and Public Health Sciences in the Department of Health Sciences at the University of Leicester, UK

Collaborators

Prof David Nicol – Consultant Urologist, Royal Marsden NHS Foundation Trust, UK

Mr Chris Ogden – Consultant Urologist, Royal Marsden NHS Foundation Trust, UK

Mr Alan Thompson – Consultant Urologist, Royal Marsden NHS Foundation Trust, UK

Prof Christopher Woodhouse – Consultant Urologist, Royal Marsden NHS Foundation Trust, UK

BARCODE 1 Collaborators: GP practices

Clinical Research Network (CRN)	Lead GP	Practice
CRN Kent, Surrey & Sussex	Dr Louise Wilkes	Ball Tree Surgery
	Dr Nigel Bird	Beaconsfield Medical Practice
	Dr Suthan Ulakanathan	Birchwood Medical Practice
	Dr Nigel Bird	Brighton Health and Wellbeing Centre

	Dr Liz Evans	Cathedral Medical Practice
	Dr Nigel Bird	Charter Medical Centre
	Dr Matthew Clark	Cranleigh Medical Practice
	Dr N'Jaimeh Asamoah-Owusu	Crouch Oak Family Practice
	Dr Tariq Hussain	Downs Way Medical Practice
	Dr Daniel Moore	Faversham Medical Practice
	Dr David Ratcliffe	Fort House Surgery
	Dr Paul Vinson	Furnace Green Surgery
	Dr Purnima Sharma	Gravesend Medical Centre
	Dr Karen Crawford Clarke	Henfield Medical Centre
	Dr Imran Rafi	Longcroft Clinic
	Dr Alison Esslemont	Maywood Health Care Centre
	Dr Louise Wilkes	New Pond Row Surgery
	Dr Vanessa Short	Newton Place Surgery
	Dr Jo Monjardino	Northbourne Medical Centre
	Dr Mei Ling Lancashire	Park House Surgery
	Dr Vanessa Short	Park Surgery - Herne Bay
	Dr Cathie Shipton	Park Surgery - Horsham
	Dr Sandeep Mtharu	Parklands Surgery
	Dr Priya Ganeshkumar	Pound Hill Medical Group
	Dr Nigel Bird	Preston Park Surgery
	Dr Liam Byrne	Rowe Avenue Surgery
	Dr Raj Sharma	Sea Road & Pebsham Surgeries
	Dr Shavetha Vasdev	Seaford Medical Practice
	Dr Alison Parrish	Selsey Medical Practice
	Dr Munira Mohamed	Sheerwater Health Centre
	Dr Suthan Ulakanathan	Smallfield Surgery
	Dr Pippa Lally	Southbourne Surgery
	Dr Marian English	St Lawrence Surgery
	Dr Nigel Bird	Stanford Medical Centre
	Dr Richard Dunn	The Churchill Clinic
	Dr Louise Wilkes	The Manor Practice
	Dr Jitesh Sahu	The Om Medical Centre
	Dr Suthan Ulakanathan	Townhill Medical Practice
	Dr Paul Deffley	Trinity Medical Centre
	Dr Nigel Bird	Warmdene Surgery
	Dr Abiola Idowu	White Cliffs Medical Centre
	Dr Richard Brice	Whitstable Medical Practice
	Dr Reema Patel	Wigmore Medical Centre
	Dr Mark Lee	Willow Green Surgery
	Dr Ruth Danson	Woodbridge Hill Surgery
CRN Thames Valley & South Midlands	Dr Jonathan Crawshaw	Berinsfield Health Centre
	Dr Christine A'Court	Broadshires Health Centre
	Dr Zishan Ali	Burma Hill Practice
	Dr Matthew J Wallard	Gosford Hill Medical Centre

	Dr Neetul Shah	Iver Medical Centre
	Dr Alison Law	Montgomery House Surgery
	Dr Chris Davies	Norden House Surgery
	Dr Christopher Davies	Stewkley Road Surgery & Wing Surgery
	Dr Grace Ding	The Chiltern Surgery/Chalgrove & Watlington Surgeries
	Dr Kathryn Brown	The Leys Health Centre/Blackbird Leys Health Centre
	Dr Tracey Largent	The Swan Practice
	Dr Suneth Godagama	Westcroft Health Centre
	Dr Joseph Rizzo-Naudi	Whitchurch Surgery
	Dr Victoria Glover	White Horse Medical Practice
	Dr Zishan Ali	Wokingham Medical Centre
CRN South London	Dr Shobana Sathananthan	Bridgstock & South Norwood Partnership
	Dr Van Den Berk	Clapham Park Group Practice
	Dr Marilyn Graham	Fairview Medical Centre
	Dr Satinder Kumar	Hurley & Riverside Clinic
	Dr Affie Etok	The Jenner Practice
	Dr Meenu Mittal	Open Door Surgery
	Dr Imtiaz Ahmed	The South Lambeth Road Practice
	Dr Lindsey Roberts	Wallington Family Practice
	Dr Michael Choong	Woodlands Practice

Figure S1Study design

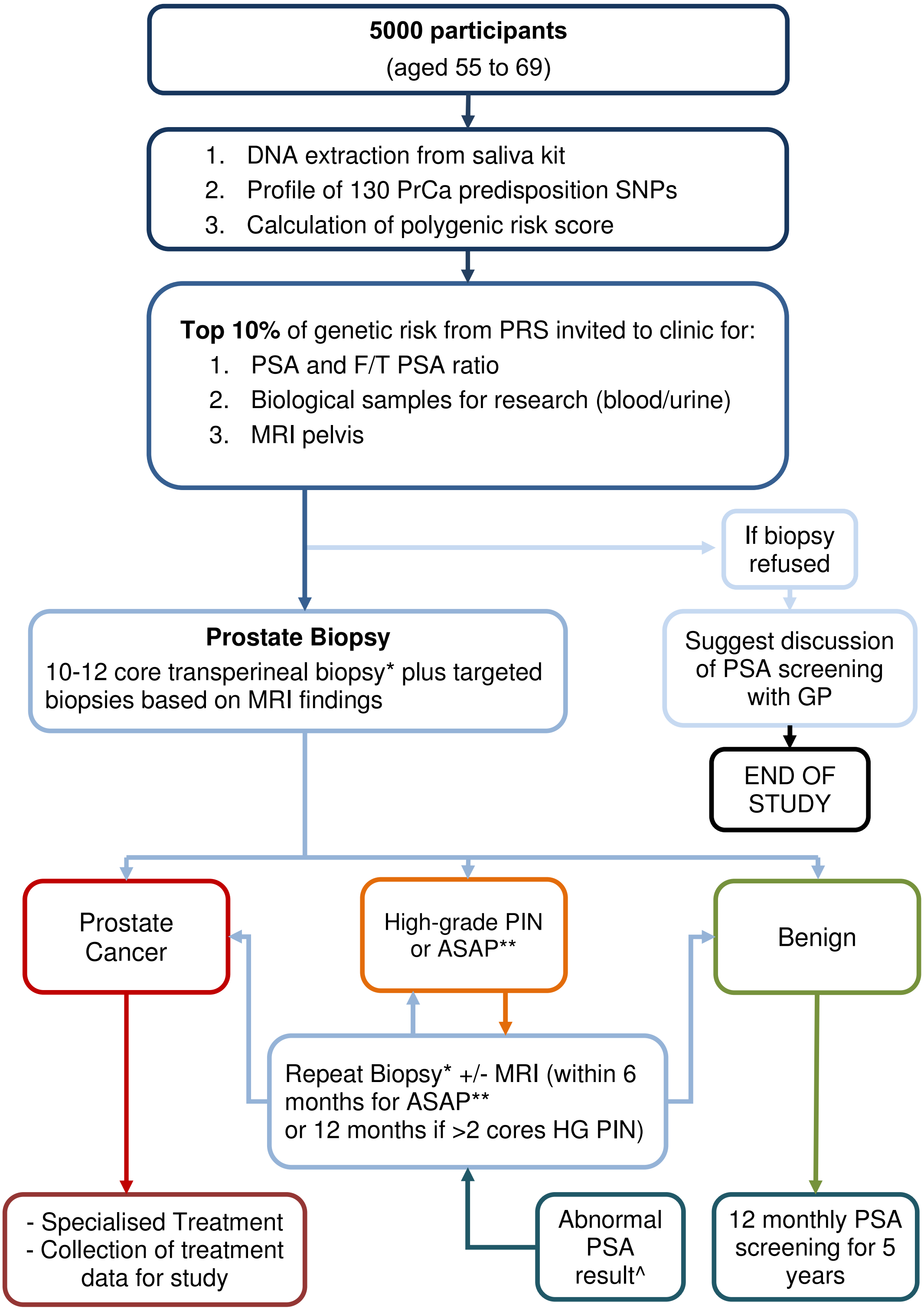

^ Abnormal PSA is >3ng/ml or a >50% increase if last reading was >3ng/ml with normal biopsy
*Template biopsy may be recommended in some cases
**ASAP: Atypical small acinar proliferation, High-grade PIN: Prostate Intraepithelial Neoplasia

Table S1: Eligibility Criteria

**Inclusion Criteria:**
• Men aged 55 to 69 years.
• Caucasian ethnicity.
• WHO performance status 0-2
• Absence of any psychological, familial, sociological or geographical situation potentially hampering compliance with the study protocol and follow-up schedule.
**Exclusion criteria**
• Non-Caucasian ethnicity (including mixed race or Ashkenazi Jewish (excluded as these groups have different genetic risk profiles from those being studied).
• Previous diagnosis of cancer with a life-expectancy of less than five years.
• Negative prostate biopsy within one year before recruitment.
• Previous diagnosis of prostate cancer.
• Co-morbidities making prostate biopsy risk unacceptable (anticoagulants or antiplatelet medication including Warfarin, Clopidogrel, Apixaban, Dabigatran or other NOAC medications (Novel Oral Anti-Coagulant); poorly controlled diabetes, cardiovascular/respiratory disease, immunosuppressive medication or splenectomy)
• Men with body mass index (BMI) 40 and above.
• Men with BMI 35 and above plus other co-morbidities.
• Contraindications to having an MRI (pacemakers, aneurysm clips, metallic cardiac valve/stent, Ventriculo-Peritoneal (VP) shunt, cochlear implant, neurotransmitter, metallic foreign bodies in eye(s), other metalwork, claustrophobia).
• Any significant psychological conditions that may be worsened or exacerbated by participation in the study.

Table S2: List of 130 Risk SNPs used to calculate PRS

rsID	Chromosome	hg19 position
rs17599629	1	150658287
rs1218582	1	154834183
rs1043608	1	153909069
rs56391074	1	88210715
rs4245739	1	204518842
rs721048	2	63131731
rs10187424	2	85794297
rs12621278	2	173311553
rs7584330	2	238387228
rs3771570	2	242382864
rs62106670	2	8597123
rs74702681	2	66652885
rs11691517	2	111893096
rs34925593	2	174234547
rs59308963	2	202123479
rs9287719	2	10710730
rs13385191	2	20888265
rs1465618	2	43553949
rs2660753	3	87110674
rs7611694	3	113275624
rs10934853	3	128038373
rs6763931	3	141102833
rs10936632	3	170130102
rs1283104	3	106962521
rs142436749	3	169093100
rs10009409	4	73855253
rs1894292	4	74349158
rs17021918	4	95562877
rs7679673	4	106061534
rs76551843	5	169172133
rs4976790	5	177968915
rs2242652	5	1280028
rs12653946	5	1895829
rs2121875	5	44365545
rs12665339	6	30601232
rs9296068	6	32988695
rs4713266	6	11219030
rs7767188	6	30073776
rs3096702	6	32192331
rs3129859	6	32400939
rs1983891	6	41536427
rs2273669	6	109285189
rs339331	6	117210052
rs1933488	6	153441079
rs9364554	6	160833664
rs17621345	7	40875192
rs11452686	7	20414110
rs12155172	7	20994491
rs10486567	7	27976563
rs56232506	7	47437244
rs6465657	7	97816327
rs183373024	8	128104117
rs2928679	8	23438975
rs11135910	8	25892142
rs12543663	8	127924659
rs10086908	8	128011937
rs16901979	8	128124916
rs620861	8	128335673
rs6983267	8	128413305
rs1447295	8	128485038
rs1048169	9	19055965
rs1182	9	132576060
rs17694493	9	22041998
rs61830900	10	871481
rs1935581	10	90195149
rs76934034	10	46082985
rs10993994	10	51549496
rs3850699	10	104414221
rs4962416	10	126696872
rs61890184	11	7547587
rs2277283	11	61908440
rs11290954	11	76260543
rs1800057	11	108143456
rs138466039	11	125054793
rs878987	11	134266372
rs1881502	11	1507512
rs7127900	11	2233574
rs7931342	11	68994497
rs11568818	11	102401661
rs11214775	11	113807181
rs2066827	12	12871099
rs10845938	12	14416918
rs7968403	12	65012824
rs7295014	12	133067989
rs5799921	12	90160530
rs80130819	12	48419618
rs10875943	12	49676010
rs902774	12	53273904
rs1270884	12	114685571
rs1004030	14	23305649
rs11629412	14	37138294
rs8008270	14	53372330
rs7141529	14	69126744
rs8014671	14	71092256
rs4924487	15	40922915
rs33984059	15	56385868
rs201158093	16	82178893
rs28441558	17	7803118
rs2680708	17	56456120
rs684232	17	618965
rs11649743	17	36074979
rs4430796	17	36098040
rs138213197	17	46805705
rs11650494	17	47345186
rs1859962	17	69108753
rs8093601	18	51772473
rs28607662	18	53230859
rs12956892	18	56746315
rs10460109	18	73036165
rs7241993	18	76773973
rs11666569	19	17214073
rs118005503	19	32167803
rs61088131	19	42700947
rs8102476	19	38735613
rs11672691	19	41985587
rs2735839	19	51364623
rs11480453	20	31347512
rs6126982	20	52456445
rs12480328	20	49527922
rs2427345	20	61015611
rs6062509	20	62362563
rs1041449	21	42901421
rs9625483	22	28888939
rs9623117	22	40452119
rs5759167	22	43500212
rs5945619	X	51241672
rs2807031	X	52896949
rs5919432	X	67021550
rs17321482	X	11482634
rs2405942	X	9814135

Table S3– Reasons for 277 persons declining / withdrawing from 745 identified in the ≥90th centile

**Withdrawal after genetic results and before MRI n=194**			
Personal Choice	125	No reason / non-responders to screening invite	53
		Refused biopsy	26
		Too far to travel/work/caring commitments	31
		Confounding health condition	12
		Already undergoing screening via primary care	2
		Concerned about risk from MRI contrast	1
Study Team Advice	65	Confounding health condition	55
		Diagnosed with Prostate Cancer	8
		Biopsy elsewhere	1
		Refused to comply with hospital Covid precautions	1
Deceased	4		
**Withdrawal after MRI and before biopsy n=83**			
Personal Choice	52	Refused biopsy	46
		No reason provided	2
		Moved away/travel/caring responsibilities	3
		Confounding health condition	1
Study Team Advice	30	Urological investigations required	19
		Confounding health condition	9
		Couldn’t tolerate biopsy	2
Deceased	1		

Table S4: Sociodemographic characteristics of those in the top 10% of PRS

**Ethnicity**	
White European	468 (100%)
**Age Years**	
Mean	61.2
Range	55-69
**Education**	
None	19 (4.0%)
School to 16	73 (15.6%)
School to 18	58 (12.4%)
Technical	96 (20.5%)
Degree / post grad	203 (43.4%)
Missing	19 (4.1%)
**Employment**	
Currently employed	281 (59.9%)
Not currently employed	9 (1.9%)
Retired	169 (36.1%)
Missing	9 (1.9%)
**Socioeconomic classification:**	
Professional/Managerial	267 (57.1%)
Skilled Manual/Non-manual	103 (22.0%)
Partly skilled manual/unskilled	48 (10.3%)
Missing	50 (10.7%)
**Family History (first or second degree relative)**	
Yes	98 (20.9%)
No	370 (79.1%)

Table S5: Breakdown of cancers stratified by PSA level

	PSA, N (%)											
NCCN	0 - ≤1.5		>1.5 - ≤3		>3 - ≤4		>4		Total		Med.	(IQR)
Low	43	(66.2)	24	(45.3)	7	(31.8)	10	(21.3)	84	(44.9)	1.0	(0.7, 1.2)
Int. Fav.	19	(29.2)	20	(37.7)	9	(40.9)	15	(31.9)	63	(33.7)	2.2	(1.8, 2.5)
Int. Unfav.	2	(3.1)	7	(13.2)	5	(22.7)	14	(29.8)	28	(15.0)	3.5	(3.3, 3.8)
High	1	(1.5)	2	(3.8)	1	(4.5)	8	(17.0)	12	(6.4)	6.5	(4.7, 9.5)
Total	65		53		22		47		187		2.1	(1.3, 4.2)

	PPV	NPV	Sens	Spec	Correctly classified	AUC	(95% C.I.)
Any cancer							
Whole cohort	40.0						
PSA	61.1	66.8	36.9	84.3	65.4	0.66	(0.61, 0.71)
MRI	62.9	66.2	32.8	87.2	65.5	0.62	(0.56, 0.67)
PSA or MRI	57.5	70.4	53.5	73.7	65.6		
PSA and MRI	83.3	63.8	16.1	97.9	65.2	0.69	(0.64, 0.74)
Clin. Sig. cancer (Gleason grade 4)							
Whole cohort	22.0						
PSA	46.0	85.6	50.5	83.3	76.1	0.75	(0.70, 0.81)
MRI	46.4	84.6	44.1	85.8	76.7	0.70	(0.63, 0.76)
PSA or MRI	39.7	88.4	67.0	71.2	70.3		
PSA and MRI	77.8	82.8	27.5	97.8	82.4	0.78	(0.73, 0.84)

Table S6: Summary of positive (PPV) and negative predictive values (NPV), sensitivity and specificity and area under the curve (AUC)

Table S7 – Univariable and multivariable models

		Any cancer				Clinically significant cancer			
		OR	(95% C.I.)	AUC	(95% C.I.)	OR	(95% C.I.)	AUC	(95% C.I.)
**Univariable models**									
Age at biopsy		1.02	(0.98, 1.07)	0.53	(0.48, 0.58)	1.06	(1.00, 1.11)	0.57	(0.50, 0.63)
Family History		1.29	(0.81, 2.05)	0.52	(0.48, 0.56)	1.99	(1.20, 3.32)	0.56	(0.51, 0.61)
PSA		1.34	(1.20, 1.48)	0.67	(0.62, 0.72)	1.45	(1.30, 1.62)	0.75	(0.70, 0.81)
PSA density		1.01	(1.01, 1.02)	0.69	(0.64, 0.74)	1.02	(1.01, 1.02)	0.76	(0.71, 0.82)
PIRADS									
	1	0.27	(0.03, 2.25)			0.78	(0.09, 6.47)		
	2	Ref.				Ref.			
	3	1.52	(0.80, 2.88)			1.45	(0.66, 3.18)		
	4	4.22	(1.94, 9.20)			6.19	(2.92, 13.12)		
	5	19.19	(4.41, 83.45)	0.61	(0.57, 0.65)	34.61	(9.91, 120.85)	0.67	(0.61, 0.72)
PRS		1.63	(0.86, 3.06)	0.54	(0.49, 0.59)	1.98	(0.98, 4.03)	0.55	(0.49, 0.61)
**Multivariable models: Age + Family History + (one of PSA, PIRADS, PRS)**									
PSA		1.33	(1.20, 1.48)	0.66	(0.61, 0.71)	1.44	(1.29, 1.61)	0.75	(0.70, 0.81)
PIRADS		1.24	(0.76, 2.03)			2.15	(1.22, 3.80)		
	1	0.28	(0.03, 2.28)			0.82	(0.10, 6.87)		
	2	Ref.				Ref.			
	3	1.55	(0.81, 2.95)			1.54	(0.69, 3.42)		
	4	4.06	(1.85, 8.89)			5.56	(2.57, 12.04)		
	5	18.81	(4.31, 82.00)	0.62	(0.56, 0.67)	35.13	(9.95, 124.09)	0.70	(0.63, 0.76)
PRS		1.70	(0.90, 3.21)	0.55	(0.50, 0.60)	2.24	(1.08, 4.65)	0.61	(0.55, 0.67)
**Multivariable models: Age + Family History + (two of PSA, PIRADS, PRS)**									
PSA		1.30	(1.16, 1.46)			1.40	(1.25, 1.58)		
PIRADS									
	1	0.19	(0.02, 1.71)			0.56	(0.06, 5.14)		
	2	Ref.				Ref.			
	3	1.27	(0.64, 2.50)			1.04	(0.42, 2.56)		
	4	3.14	(1.38, 7.17)			4.09	(1.74, 9.61)		
	5	10.72	(2.36, 48.82)	0.69	(0.64, 0.74)	19.58	(5.17, 74.09)	0.78	(0.73, 0.84)
PIRADS									
	1	0.26	(0.03, 2.17)			0.76	(0.09, 6.41)		
	2	Ref.				Ref.			
	3	1.54	(0.81, 2.93)			1.52	(0.68, 3.38)		
	4	3.93	(1.79, 8.64)			5.32	(2.43, 11.62)		
	5	18.23	(4.17, 79.58)			33.58	(9.49, 118.80)		
PRS		1.48	(0.74, 2.94)	0.62	(0.57, 0.67)	1.76	(0.77, 4.04)	0.70	(0.64, 0.76)
PRS		1.26	(0.64, 2.50)			1.45	(0.63, 3.32)		
PSA		1.33	(1.20, 1.48)	0.66	(0.61, 0.71)	1.44	(1.29, 1.60)	0.75	(0.70, 0.81)
**Multivariable models: Age + Family History + PSA + PIRADS + PRS**									
PRS		1.23	(0.60, 2.51)			1.30	(0.53, 3.20)		
PSA		1.30	(1.16, 1.45)			1.40	(1.24, 1.57)		
PIRADS									
	1	0.19	(0.02, 1.67)			0.55	(0.06, 4.98)		
	2	1.00	(1.00, 1.00)			1.00	(1.00, 1.00)		
	3	1.27	(0.64, 2.50)			1.03	(0.42, 2.55)		
	4	3.10	(1.35, 7.08)			4.00	(1.69, 9.45)		
	5	10.70	(2.35, 48.76)	0.69	(0.64, 0.74)	19.48	(5.14, 73.75)	0.78	(0.73, 0.84)

Table S8: Estimates of overdiagnosis

**Scenario 1: PRS as the screening test**							
**PRS only**							
	**Average age**	**% GS6**	**%GS 7**	**%GS>7**	**% of total cancers**	**Estimated mean sojourn time**	**% Overdiagnosis**
55-59	58.2	0.44	0.56	0.00	0.18	10.3	9.7
60-64	61.8	0.57	0.43	0.00	0.36	12.2	18.3
65-69	66.9	0.31	0.65	0.04	0.29	11.5	23.0
70-74	70.9	0.44	0.50	0.06	0.17	12.9	33.9
**Weighted average**							**20.8**
**Scenario 2: Screening with MRI men >90th centile of risk distribution (PIRADS ≥3 is considered positive test result)**							
	**Average age**	**% GS6**	**%GS 7**	**%GS>7**	**% of total cancers**	**Estimated mean sojourn time**	**% Overdiagnosis**
55-59	58.0	0.33	0.67	0.00	0.20	9.3	7.0
60-64	61.9	0.56	0.44	0.00	0.26	12.2	18.0
65-69	67.2	0.10	0.76	0.14	0.34	9.0	15.0
70-74	70.7	0.08	0.83	0.08	0.20	8.8	21.0
**Weighted average**							**15.6**
**Scenario 3: Screening with PSA men ≥90th centile of risk distribution (PSA ≥3 is considered positive test result)**							
	**Average age**	**% GS6**	**%GS 7**	**%GS>7**	**% of total cancers**	**Estimated mean sojourn time**	**% Overdiagnosis**
55-59	58.2	0.17	0.83	0.00	0.08	7.7	4.0
60-64	62.0	0.28	0.72	0.00	0.34	9.5	11.0
65-69	67.0	0.26	0.70	0.04	0.37	10.8	21.0
70-74	70.8	0.20	0.67	0.13	0.21	10.2	25.0
**Weighted average**							**17.2**

Supplementary Information 1: Overdiagnosis calculations

We estimated the probability of overdiagnosis as the probability that screen-detected cancer would have taken longer than the remaining lifetime to progress to clinical cancer[25], as

$$\int_t^\infty \lambda e^{-\lambda x}\, dx = e^{-\lambda t}$$

Where t is the expected remaining lifetime, and λ is the rate of progression from preclinical to clinical cancer. Under the exponential distribution, $1/\lambda$ is the mean sojourn time (MST).

We used estimates of MSTs from published literature, based on data from prevalence screening in the ProtecT and Cluster Randomised Trial of PSA Testing for Prostate Cancer (CAP) [REF: Martin et al 2024 https://jamanetwork.com/journals/jama/fullarticle/2817322]. In Martin et al, the age-specific overall MSTs were derived using multistate survival model. In Pashayan et al [26], age-specific MST for GS≥7 were calculated as the number of years where the cumulative incidences catches up with the preclinical incidence. MST for GS < 7 was derived as

$$\frac{MST_{overall} - (\, MST_{GS\geq 7} * proportion_{GS\geq 7})}{proprtion_{GS<7}}$$

Age-specific proportions of tumours with GS < 7 and GS≥7 were based on ProtecT.

Supplementary Table 8 gives the MST estimates and Supplementary Table 9 the expected remaining lifetime

Table S9: Mean sojourn time estimates by Gleason score and age groups

Age group	MST GS < 7 (years)	MST GS ≥7 (years)
50-54	13.88	6.88
55-59	15.68	5.65
60-64	16.53	6.19
65-69	19.90	6.69

Table S10: Expected remaining lifetime by age for men in the UK, 2020-2022

Age	Remaining life expectancy (years)
50	30.97
51	30.08
52	29.20
53	28.32
54	27.45
55	26.59
56	25.73
57	24.88
58	24.03
59	23.19
60	22.36
61	21.55
62	20.74
63	19.94
64	19.15
65	18.37
66	17.60
67	16.85
68	16.10
69	15.37
70	14.65
71	13.93
72	13.23
73	12.55
74	11.87

Table S11: Diversity and Representativeness Table: demographics of the UK population affected by prostate cancer

Category	
Disease under investigation	Prostate Cancer (PrCa)
Special considerations related to	
Sex + Gender	PrCa affects people born with prostates (those assigned male sex at birth)
Age	PrCa incidence increases with increasing age. In this study we included those aged 55-69 years.
Race/ancestry/ethnic group	In the United Kingdom PrCa affects 1 in 8 of those of European ancestry, 1 in 4 of those of African or African Caribbean ancestry and 1 in 13 of those of Asian ancestry. At the time of study development, the only validated single nucleotide polymorphisms (SNPs) to generate a polygenic risk score (PRS) were in those of European ancestry, while data were awaited on those of diverse ancestries.
Geography	At present there are no global guidelines for PrCa population screening unlike policies in place for other common solid tumours e.g. Breast Cancer, Colon Cancer, while worldwide the incidence of PrCa increases.
Other considerations	While differences exist between the UK population and that of other countries this study is the first of its kind to evidence the usefulness of PRS in PrCa screening to help identify those at high risk of developing PrCa and to make a case for targeting screening programmes to those most at risk in a population.
Overall representativeness of this study	The participants in the current study self-reported gender, age and ancestry.<br>We recognise the lack of ancestral diversity in the participants in the current study as a limitation.<br>This study demonstrates proof of principle of utilising a polygenic risk score to risk stratify for PrCa in those of European ancestry as these were the validated SNPs used at the time of the study development.<br>This can now be expanded to include those of more diverse ancestry given the more recently discovered and validated SNPs in multi-ancestry laboratory work.

Table S12: Description of all adverse events in the BARCODE1 Study

Description of Adverse Event	Number of persons affected	Outcome
Syncope post-phlebotomy	n =1	transient
Syncope post-biopsy	n = 2	transient
Syncope post-cannulation	n = 1	transient
MRI done without contrast	n = 1	MRI was double-read
Incidental bladder finding on MRI	n = 2	Treated for incidental finding
Urinary tract infection	n = 2	Antibiotics oral
Sepsis	n = 1	Antibiotics intravenous
Catheterisation post-biopsy	n = 1	transient

Supplementary Information 2: Participant questionnaire used to collect demographic and health information

BARCODE 1 STUDY ELIGIBILITY QUESTIONNAIRE

Please tick the appropriate box/es:

**1.** To which of these do you consider yourself to belong?	
**White**<br>☐ White British<br>☐ Any other White: ______________________<br>**Jewish**<br>☐ Ashkenazi<br>☐ Sephardi<br>☐ Mixed<br>**Mixed**<br>☐ White and Black Caribbean<br>☐ White and Black African<br>☐ White and Asian<br>☐ Any other Mixed: ______________________	**Black or Black British**<br>☐ Caribbean<br>☐ African<br>☐ Any other Black: ______________________<br>**Asian or Asian British**<br>☐ Indian<br>☐ Pakistani<br>☐ Bangladeshi<br>☐ Any other Asian: ______________________<br>☐ **Chinese**<br>☐ **Any other:** ___________________________

2. Have you ever been diagnosed with cancer? ☐ No ☐ Yes *(please specify below)*

Cancer site/s: ____________________ Age of diagnosis: __________

Details and date of last treatment/s: ____________________

____

____

____

3. Have any members of your family been diagnosed with prostate cancer? ☐ No ☐ Unknown ☐ Yes (*please specify below)*

a Relative 1 Relationship: ____________________ Age: ______

b Relative 2 Relationship: ____________________ Age: ______

c Relative 3 Relationship: ____________________ Age: ______

d Relative 4 Relationship: ____________________ Age: ______

e Relative 5 Relationship: ____________________ Age: ______

4. Do you have a family history of any cancer in your first or second degree relatives?
Please only include cancers in parents, siblings, children, grandparents, aunts, uncles, grandchildren, nieces or nephews ☐ No ☐ Unknown ☐ Yes *(please specify below)*

a Relative 1 Rel'ship: __________ Cancer site: __________ Age: ______

b Relative 2 Rel'ship: __________ Cancer site: __________ Age: ______

c Relative 3 Rel'ship: __________ Cancer site: __________ Age: ______

d Relative 4 Rel'ship: __________ Cancer site: __________ Age: ______

e Relative 5 Rel'ship: __________ Cancer site: __________ Age: ______

f Relative 6 Rel'ship: __________ Cancer site: __________ Age: ______

g Relative 7 Rel'ship: __________ Cancer site: __________ Age: ______

Please note that we are gathering family history information for research purposes only. If you have a significant family history of cancer, you may wish to discuss this with your GP and they may recommend a referral for a formal genetic assessment.

5. Do you or have you ever suffered with any of the following?

a Heart problems ☐ No
☐ Yes Specify: ____________________

b Stroke ☐ No
☐ Yes Date: ____________________

c Kidney problems ☐ No
☐ Yes Specify: ____________________

d Type 2 Diabetes ☐ No
☐ Yes Last HBA1C date and value (if known): ____________________

6. Do you take regular blood thinning medications (e.g. Aspirin, Warfarin, Clopidogrel, Heparin Rivaroxaban, Betrixaban, Darexaban or Apixaban)?

☐ No

☐ Yes Specify medication: ____________________

Number of years taken: __________ Dosage: ____________________

7. Do you take regular diabetes medication (e.g. metformin)?

☐ No

☐ Yes Specify medication: ____________________________

Number of years taken: ________________ Dosage: ______________________

8. Have you ever had a biopsy of the prostate gland?

☐ No

☐ Yes Date: _______________________ Reason: _________________________________

9. Have you ever had a PSA test?

☐ No

☐ Unknown

☐ Yes Date: _______________________ Value (if known): __________________________

10. Have you ever had any problems with and /or investigations on your prostate gland (e.g. prostatitis, enlarged prostate)?

☐ No

☐ Unknown

☐ Yes Specify: ____

11. What other medication are you currently taking?

____

____

____

____

____

____

____

12. Do you suffer from any medical conditions not listed above?

____

____

____

____

____

____

____

Demographic Data

13. What is your employment status?

☐ In active paid work
☐ Retired doing voluntary work
☐ Retired
☐ Unemployed and seeking work
☐ Unemployed due to illness or disability

14. What is your current / last job title: ____

15. What is your highest level of qualification?

☐ O Level / GCSE
☐ A Level
☐ HND / BTEC
☐ NVQ
☐ Degree
☐ Post graduate
☐ None
☐ Other: ___________________________

16. Do you smoke?

☐ No

☐ Yes, currently — Number of years smoked: ___________________ Approximate number of cigarettes per day ____________

☐ No, ex-smoker — Number of years smoked: ___________________ Approximate number of cigarettes per day ____________

17. Alcohol consumption (per week): ____________________

Please specify the approximate number of units of alcohol you drink per week (1 unit is equal to half a pint of beer, a small glass of wine or one measure of a spirit)

18. Please enter your height: ________ feet _______ inches OR __________ metres

19. Please enter your weight: ________ stone _______ lbs OR ___________ kgs
